# Our Exchanges of 1845

**Published:** 1912-05

**Authors:** 


### OUR CONTEMPORARIES

### Our Exchanges of 1845

DURING the first year of existence of the BUFFALO MEDICAL JOURNAL (1845), the editor, Dr. Austin Flint, printed a brief description of each of his exchanges, covering all of the American medical journals known to him. It may be of interest to reproduce his description.

The *American Journal of the Medical Sciences*, the oldest, in existence 26 years at that time, published quarterly, at least 264 pages, $5.00 per annum, Lea & Blanchard, accompanied by a monthly of 32 pages devoted to general medical intelligence and republication of standard works. This is still in existence, as a monthly of the highest order.

Other Philadelphia journals were: *Bulletin of Medical Sciences,* 35 pages, monthly, published in connection with the

Select Medical Library. *Medical Examiner, Journal of Pharmacy, Journal and Library of Dental Science, Stockton's Dental Intelligencer.* Details are given only of the first and last, 35 and 24 pages respectively, both monthly and both costing a dollar a year.

*New Orleans Medical and Surgical Journal,* bi-monthly, 140 pages, $5.00 per annum. "This is one of our ablest contemporaries," a remark which, without the quotation marks, holds good in 1912. This journal was established the year before the BUFFALO MEDICAL JOURNAL. At present it is a monthly of about 85 pages.

*Southern Medical and Surgical Journal,* Augusta, Ga., 64 pages, monthly, $3.00 per annum, edited by Drs. Eve and Garvin.

*Southern Journal of Medicine and Pharmacy,* Charleston, bi-monthly, 120 pages, $4.00 per annum, established in 1845.

*Western Journal of Medicine and Surgery,* Louisville, monthly, 90 pages, $5.00.

*Western Lancet,* Lexington, Ky., monthly, 50 pages, $3.00.

*St. Louis Medical and Surgical Journal,* monthly, 40 pages, $3.00. *Missouri Medical and Surgical Journal,* monthly, 24 pages, $2.00, also published in St. Louis.

*Illinois Medical and Surgical Journal,* Chicago, monthly, 16 pages, $1.00.

*The Boston Medical Journal* "is a weekly or monthly as its patrons elect." (Apparently, this means that subscriptions were taken for one issue a month). It was the only weekly in the country, $3.00. It is also the oldest journal in America, except the *American Journal of the Medical Sciences.*

*Journal of Health,* Boston, monthly, 24 pages, $1.00, established 1845.

*American Journal of Insanity, Utica,* 96 pages, quarterly, $1.00.

Our distinguished predecessor alludes to Philadelphia as "hitherto if not still, the Medical Paris of America." "New York has hitherto been unfortunate in her medical periodicals; those which have at successive periods been established, have been short lived." Only two are mentioned as extant: *New York Journal of Medicine* and *Collateral Sciences,* six years old, bi-monthly, 144 pages, $3.00; *Medical and Surgical Reporter,* bi-weekly, 16 pages, $2.00.

The foregoing are stated to be all of the medical publications of the country with which the editor is acquainted, even by title. A single Canadian journal is mentioned: *British-American Journal of Medical and Physical Science, monthly,* 27 pages, $3.00, lately sprung from the ashes of the *Montreal Gazette.*

At present, according to H. R. Harrower's compilation, there are 123 regular medical journals on general medicine, 65 on specialties, and about 150 others, variously classified and bearing more or less on medicine.

With due allowance for errors in compilation, it appears that the BUFFALO MEDICAL JOURNAL ranks fourth in age, for America, being antedated by the *American Journal of the Medical Sciences, the Boston Medical and Surgical Journal* and, by a few issues, by the *New Orleans Medical and Surgical Journal.*

---

THE *American Practitioner and News,* published for forty-five years in Louisvile, Ky., and *The New England Medical Monthly* with *The Annals of Medical Practice,* established in Boston, since 1888, are now combined into *The American Practitioner,* issued monthly from No. 80 Washintgon Square, East, New York City, under the editorship of John W. Wainwright.

---

AT the risk of being accused of possessing a specialist's myopia, we recommend to all our readers, the *American Journal of Gastro-enterology* published quarterly in Philadelphia, and Edited by Dr. Lewis Brinton. We may certainly disclaim anything more than a friendly interest, as our connexion with that journal is purely honorary.

---

IN June the *American Journal of Surgery* will issue a number composed of original contributions from men of recognized prominence in the medical profession residing in Greater New York. Among those to contribute are Herman J. Boldt, C. U. Dowd, Meddaugh Dunning, Wm. S. Gottheil, E. L. Keys, Jr., Howard Lilienthal, Chas. H. May, Willy Meyer, Robert T. Morris, S. Lewis Pilcher, John O. Polak, James P. Tuttle, James P. Warbasse and others.

---

WE do not as a rule endorse therapeutic suggestions from laymen, nor do we believe in spring tonics but this, from the *Farm Journal* of Philadelphia is about the best we have seen:

An excellent spring tonic, after several months of hovering around the fireside, is a good sawbuck, a sharp saw and a pile of hard wood. We know it for we have tried it.

---

IN an article on the treatment of sick children in the March *Woman's Home Companion,* the author Dr. Roger H. Dennett, a famous New York specialist on the disease of children says:

"Never, never, expose the child to any contagious disease in order that he may have it once and be done with it. Even the so-called simple children's disease, such as measles or whooping-cough, have a death-rate that is appalling."

We would call the attention of our readers to the great practical value of the State and Municipal Bulletins. These are—or will be— sent to every physician so that we need not refer to them in detail.

---

FOR SALE: A perfectly good though much used copy of Mrs. Eddy's book. Presented by the former owner to her physician at the successful termination of a claim in the ear, the error taking the material form of pus. Neither the present nor the former owner has any further use for it but can endorse it in the highest terms since, under its influence, sickness was non-material for several years.

